# ﻿Uganda’s endemic flora: discovery, diversity, distribution and threat status

**DOI:** 10.3897/phytokeys.269.173801

**Published:** 2026-01-06

**Authors:** James Kalema, Sophie L. Richards, Samuel Ojelel, Haley Gladitsch, Hannah Wheatcroft, Henry Miller, Kennedy Mukasa, Ben Kirunda, Thomas G. Forrest, Thomas C. Cole, Isabel Baldwin, Elizabeth Downes, W. R. Quentin Luke, Iain Darbyshire

**Affiliations:** 1 Department of Plant Sciences, Microbiology & Biotechnology, Makerere University, P.O. Box 7062 Kampala, Uganda Makerere University Kampala Uganda; 2 Royal Botanic Gardens, Kew, Richmond, Surrey, TW3 9AE, UK Royal Botanic Gardens Richmond United Kingdom; 3 Total Energies EP Uganda (Tilenga Project), RR Pearl Tower One, Plot 1, Yusuf Lule Road, P.O. Box 34867 Kampala, Uganda Total Energies EP Uganda (Tilenga Project) Kampala Uganda; 4 Plot 116, Buziga Hill Road, Kampala, Uganda Unaffiliated Kampala Uganda; 5 801 Cold Spring Road, Santa Barbara, CA 93108, USA Unaffiliated Santa Barbara United States of America; 6 East African Herbarium, National Museums of Kenya, Nairobi, Kenya National Museums of Kenya Nairobi Kenya

**Keywords:** Checklist, conservation, diversity, endemism, herbaria, Important Plant Areas, range-restricted, species richness

## Abstract

Threats including habitat loss and overexploitation continue to endanger plant diversity in Uganda. Heightened effort is needed to more effectively protect threatened species to ensure ecosystem service provision and support of livelihoods. One important step in this direction is to identify areas locally rich in biodiversity in general, and endemism in particular, where conservation efforts may most effectively contribute to protecting global biodiversity. We analysed data on the distribution of 4,816 known species of native vascular plants of Uganda to identify the endemic and near-endemic plant taxa. Distribution data were obtained from herbarium records and online resources and more than 5,000 occurrence points of priority plant taxa in Uganda collated in a BRAHMS database. A total of 184 endemic and near-endemic taxa of vascular plants are identified and documented, of which 52 are strict-endemic and 82 are near-endemic species, with a further 15 strict-endemic and 35 near-endemic infraspecific taxa. These together represent 3.8% of the known vascular flora of Uganda, with families including Asparagaceae, Asphodelaceae and Asteraceae contributing significantly to the endemic flora. These endemics cover a wide range of growth forms, but the majority (60%) are herbaceous whilst tree taxa contribute less than 15% of the total endemic flora. Significant spatial variation in the richness of endemic species is observed. The regions with the highest endemism are the montane areas of the Albertine Rift and of Eastern Uganda. However, as botanical inventories extend to understudied areas, we might expect that species new to science continue to be described. More than 90% of the endemic flora of Uganda has been assessed for its extinction risk, of which 58% are currently assessed as threatened (VU, EN, or CR on the IUCN Red List). The results of this study highlight the significant risk faced by plants unique to Uganda and have helped to inform the identification of Important Plant Areas as a key step towards spatial conservation prioritisation for Uganda’s critical plant diversity and the ecosystem services it supports.

## ﻿Introduction

The Post-2020 Kunming-Montreal Global Biodiversity Framework (GBF), finalised during the 15^th^ meeting of the Conference of the Parties to the Convention on Biological Diversity in 2022, sets ambitious targets to halt and reverse biodiversity loss. These include a call for the effective conservation and management of at least 30% of the world’s terrestrial, inland water, coastal and marine areas by the year 2030, especially areas of particular importance for biodiversity and ecosystem functions and services: the “30 by 30” target (CBD 2025).

Endemism has long been recognised as a critical phenomenon in biodiversity conservation (Darbyshire et al. 2019; Walas and Taib 2022; Stone et al. 2024) and as such, is an important means of identifying areas of particular importance for biodiversity. Taxa exhibiting narrow endemism are regarded as high priority (Swenson et al. 2012; Orsenigo et al. 2018; Bamigboye 2019; Marshall et al. 2022; Abdelaal et al. 2024) and, alongside high levels of threat to biodiversity, are the basis for identification of biodiversity hotspots (Myers 1988; Reid 1998; Cañadas et al. 2014; Marchese 2015; Costello et al. 2022; Qari 2024). Endemic taxa are also important for their unique genetic material and are an irreplaceable component of ecosystems (Carbutt 2023; Zhensikbayeva 2024), with high evolutionary and sometimes economic and ecological significance in their areas of occurrence (D’Antraccoli et al. 2023; Al-sodany et al. 2024). Determining where species diversify (cradles) and persist (museums) over evolutionary time is fundamental to understanding the distribution of biodiversity and for conservation prioritisation (Borokini 2014; Dagallier et al. 2020). However, endemic taxa are often at risk of extinction owing to their restricted range, small population size and often specialised niches (Coelho et al. 2020), which render them vulnerable to local threats, including the increasing risk posed by climate change (Cho et al. 2024; Zhensikbayeva 2024).

Assessments of risk to biodiversity often rely on spatial distribution of species and ecosystems (Keith et al. 2017). The IUCN Red List of Threatened Species is widely considered to be the most authoritative and principal source of information on global extinction risk for species (Coelho et al. 2020; Nic Lughadha et al. 2020; El-Khalafy et al. 2024; Law et al. 2024). Gallagher et al. (2023) examined country-based endemic plant species globally and found that 58% of them had no conservation assessment. In the absence of such assessments, local protection frameworks of policies and laws have no obvious locus to support conservation of particular endemic plant species. Furthermore, areas of exceptional importance for plant conservation are also often suitable for agriculture, mining and other activities, making them extinction hotspots (Le Roux et al. 2019).

To be effective in biodiversity conservation, Protected Areas (PAs) and Other Effective Conservation Measures (OECMs) should reflect important biodiversity across multiple taxa, including sites with assemblages and/or unique occurrences of endemic taxa. At present, however, areas of high endemism are often poorly represented in the global PA network (Swenson et al. 2012).

### ﻿Uganda’s plant diversity and conservation challenges

Despite its small size (241,551 km^2^) relative to many African countries, Uganda is of global importance for biodiversity conservation, being the eighth most biodiverse country in Africa, with appreciable species richness in plants, birds and mammals (Plumptre et al. 2007, 2019). It has a wide variety of habitats (Langdale-Brown et al. 1964; van Breugel et al. 2014, 2015; Richards et al. 2024) across eight different ecoregions (Dinerstein et al. 2017), spanning an altitudinal range of 620 to 5,819 m a.s.l. It is also a meeting point for six of White’s (1983) African phytochoria, from the moist Guineo-Congolian forests of the west and southwest to the subarid Somalia-Masai bushlands and thickets of the northeast and the Afromontane forests and Afroalpine zones of the mountains flanking the Lake Victoria Basin.

Uganda’s plant and habitat diversity have been documented previously through the Flora of Tropical East Africa (FTEA) series (1952–2012) and the vegetation maps of Langdale-Brown et al. (1964) and van Breugel et al. (2014, 2015). However, many of the biodiversity assessments of Uganda have focussed primarily on fauna and even those that include plants have tended to focus primarily on trees (e.g., Eggeling and Dale 1952; Uganda Forest Department 1996; Kalema and Beentje 2012; Kalema and Hamilton 2020). Exceptions have tended to focus on particular plant groups, such as Phillips et al. (2003) on the grass flora and Kalema et al. (2016) on carnivorous plants. There has been no previous comprehensive study on the endemic flora of Uganda.

The main threats to plants in Uganda include loss and degradation of habitat, agricultural expansion, introduction of non-native (alien) species, over-exploitation, excessive burning and pollution (Kalema et al. 2010; NEMA 2016; Katswera et al. 2020). Different species of plants are steadily declining in abundance in the country owing to heavy exploitation for energy resources, as more than 90% of the population depends on biomass for fuel (Bamwesigye et al. 2020). The network of protected areas, especially National Parks, offers the best protection to Uganda’s flora, but there are still many species under no formal protection. As an example, in Uganda, the globally Vulnerable *Afzelia africana* Pers. is only effectively protected in Murchison Falls National Park. Uganda’s endemic and narrowly-ranged species are often highly susceptible to extinction; a clear understanding of these species, where they occur and the threats they face, is therefore critical for their conservation (Ssali et al. 2023).

In response to these threats faced by Uganda’s flora, an Important Plant Areas (IPAs) project was launched in Uganda by Makerere University and the Royal Botanic Gardens, Kew, starting in 2017 but in earnest from 2021, with a view to averting the decline and possible extinction of critical plant species and habitats nationally (see https://www.kew.org/science/our-science/projects/tropical-important-plant-areas-uganda). IPAs - a criterion-based biodiversity assessment scheme - are defined as the most important places in the world for wild plant and fungal diversity, and are identified based on the presence of (a) threatened species, (b) exceptional botanical richness and (c) threatened habitats (Darbyshire et al. 2017). Endemic species form an important subset of IPA trigger species under both (a) and (b) and so a comprehensive understanding of the endemic flora is an essential step in the IPA identification process. This initiative falls in line with Uganda’s National Biodiversity Strategy and Action Plan (NBSAP) which proposed ‘protection of threatened, endemic and vulnerable species inside and outside protected areas’, as one of the key activities during the 2015–2025 period (NEMA 2016).

### ﻿Aims of the current study

The current work aims to document in detail the endemic and near-endemic flora for which Uganda has primary custodianship, and to understand the distribution and threat status of these taxa. This is intended to support the identification of IPAs, and also trigger other conservation initiatives focused on these endemic taxa and their related habitats and sites.

## ﻿Materials and methods

### ﻿Compilation of the checklist

The checklist of endemic and near-endemic plants of Uganda was initiated by compiling a long-list of candidate taxa from the List of East African Plants (LEAP) database (Knox and Berghe 1996), which was in turn derived and updated from the Flora of Tropical East Africa (FTEA) series. FTEA (1952–2012) is the world’s largest complete tropical regional flora, covering 12,104 plant species including 202 angiosperm families (Beentje 2016). This list was supplemented by a review of FTEA volumes published since the last update of LEAP and by consulting a wide range of taxonomic literature including monographs and protologues. All species recorded only or primarily from Uganda, or from an apparently narrow cross-border range, were included in this long-list. In addition, the World Checklist of Vascular Plants (WCVP; Govaerts et al. 2021) was queried using rWCVP (Brown et al. 2023a), by searching for all accepted taxa that are recorded in the checklist only from Uganda or only from Uganda and one additional country. This list was cross-checked against the long-list and any taxa found missing were added to the list for further consideration. Finally, the International Plant Names Index (IPNI 2025) was queried for any botanical names published post-2000 with “Uganda” included in the “Distribution of types” filter to pick up any additional recently described species of relevance.

Based upon this long-list of priority species, occurrence data were compiled by staff and trained volunteers at Kew and Makerere University. Herbarium metadata were collected directly from specimens housed at BM, EA, K and MHU (herbarium codes follow Thiers, updated continuously). Additional occurrence data were derived from the literature, most notably from FTEA and from species protologues found via IPNI (2025). Digitised specimen data were also incorporated from online data repositories, primarily through the Global Biodiversity Information Facility (extracted from GBIF.org 2025). Other important online collections consulted were the herbaria of the Missouri Botanical Garden (Tropicos 2025) and Plantentuin Meise (2024). Unvouchered observation data were also incorporated when deemed to be of a reliable source. Databasing of these collections and sight records for priority species was undertaken in BRAHMS (Version 7.9), with over 5,000 Ugandan records compiled through the project, this total comprising candidate endemic and near-endemic taxa as well as threatened non-endemic taxa of relevance to the IPAs of Uganda project.

Where no geographic coordinates were provided on specimen labels, georeferencing was carried out using printed and online gazetteers including the FTEA Index of Collecting Localities (Polhill 1988), the equivalent resource for the Flora d’Afrique Centrale (Bamps 1982) and the online gazetteer GeoNames (2025). Google Earth Pro (2025) was also used to search specimen localities, refine some coordinates derived from gazetteers and view species distribution maps to spot and rectify anomalies. Where possible, an estimate of the georeference resolution was provided, allowing for imprecise coordinates to be excluded from analyses.

Using the completed occurrence dataset, each taxon on the long-list was then evaluated against the criteria for endemic and near-endemic status (see below) to derive the final checklist.

### ﻿Definitions of endemism and near-endemism

An endemic species is defined as one restricted to a particular geographic region due to a range of factors such as isolation, and response to abiotic conditions (Zhensikbayeva 2024). Taxa included in the checklist are either strictly endemic to Uganda (i.e., they only occur within its political borders, labelled E), or are cross-border “near-endemics” (NE), as defined by one or more of the following criteria:

- a majority of the taxon’s range lies within Uganda, and it is scarce and/or highly range-restricted beyond (NE1); and/or
- the global range of the taxon is less than 10,000 km ^2^ (NE2).

The aim was to include all taxa for which Uganda has a high responsibility for their global persistence. We acknowledge that no definition of “near-endemic” is perfect, but have tried to be as objective as possible when applying the criteria set out above. We have also tried to be exhaustive, but our intention is to maintain this list and publish additions and amendments as they are uncovered. The range size of 10,000 km^2^ in NE2 is aligned to relevant range size values used in criterion B(ii) of the Important Plant Areas criteria (Darbyshire et al. 2017).

Estimates of range size were based on mapping of available locality data from the BRAHMS database, using the Minimum Convex Polygon (MCP) method commonly applied in the calculation of extent of occurrence (EOO) in the criteria for the IUCN Red List of Threatened Species (Bachman et al. 2011; IUCN 2012; Joppa et al. 2016).

### ﻿Taxonomic backbone and history of species discovery

Plant family circumscription follows the Angiosperm Phylogeny Group (2016) APG IV classification for flowering plants, with the addition of the recently described Afrothismiaceae (Cheek et al. 2024a). The Pteridophyte Phylogeny Group (2016) family classification is followed for pteridophytes, and Christenhusz et al. (2011) for gymnosperms. Accepted names of species and infraspecific taxa generally follows POWO (2025). Where the taxonomic concept adopted is not universally accepted, or where a taxon has been very recently re-combined, the alternative name is given in square brackets (e.g. for species of *Sansevieria* Thunb. / *Dracaena* L.). All published endemic and near-endemic species, subspecies and varieties are included in the checklist. A list was compiled separately of undescribed taxa that are provisionally considered to be endemic or near-endemic to Uganda but are represented by incomplete material. These are presented in a separate table to the main endemics checklist.

The date of original publication is recorded for each taxon. As the aim here is to chart the discovery of Uganda’s endemic flora, it is the date of first publication of the taxon that is of importance, rather than the publication date of the currently accepted name. Hence, for taxa that have changed genus or taxonomic rank since they were first published, we record the date of publication of the basionym. For species that were described from Ugandan material, we also list the primary collector of the type material.

### ﻿Plant growth form data

Data on plant growth form and life cycle for each taxon were derived primarily from FTEA, POWO (2025) and taxonomic protologues. A simple classification with six main categories was used: tree, shrub, liana, herb, pteridophyte and cycad. The herb category was subdivided into annual (a), perennial (p), succulent-perennial (s), epiphytic-perennial (e), climbing-perennial (c), geophyte (geo) and graminoid (gram-a for annual and gram-p for perennial). Trees and shrubs also have a succulent subdivision (s). Species with variation in growth form and/or life cycle were recorded in two or more categories. This classification follows that used in Darbyshire et al. (2019).

### ﻿Taxon distribution, habitat and biogeographic affinities

The distribution of each taxon within Uganda was recorded, first from across the four FTEA floristic regions it occurs in (U1–4) and second from key localities of its occurrence in Uganda, derived from the BRAHMS database. This locality information, whilst not intended to be exhaustive, was provided to help with future study of these species, and to assist with the identification of Important Plant Areas. For near-endemic species, the other country (or countries) in which the species occurs was recorded.

A brief record of the habitat preferences of each species is provided, derived primarily from FTEA and from the BRAHMS dataset. To aid in the understanding of the biogeographical affinities of the endemic and near-endemic flora, we also denote which ecoregion(s) (Olson et al. 2001; Burgess et al. 2004; Dinerstein et al. 2017) and phytogeographical region(s) (White 1983) each species is recorded from; most species are restricted to a single ecoregion / phytochorion but some span more than one, particularly those found within White’s (1983) Lake Victoria regional mosaic, hence a species can be assigned to more than one of each category.

### ﻿Weighted endemism analysis

The georeferenced BRAHMS database was analysed to understand the spatial distribution of endemism in Uganda. Weighted endemism values for each taxon were calculated by combining the inverse of two range metrics: Area of Occupancy (AOO) and Extent of Occurrence (EOO) as defined by the IUCN Red List of Threatened Species (IUCN 2012). Further details of this calculation are given in the Suppl. material 1. Weighted endemism was then totalled for all species occurring within a cell in a grid of hexagonal cells of quarter degree height. These data were mapped alongside taxon richness of endemic and near-endemic species per cell. Data analysis and mapping were performed in R (Wickham 2016; Pebesma 2018; Wilke 2020; R Core Team 2023; Wickham et al. 2023).

### ﻿Extinction risk and the IUCN Red List

Global extinction risk assessments were recorded for all taxa in the checklist that have been assessed to date using the categories and criteria of the IUCN Red List of Threatened Species (IUCN 2012). The assessments cited are either published on the IUCN Red List (IUCN 2025) or, where marked with an asterisk (*), have been reviewed and are “in press”, pending publication. A total of 93 endemic and near-endemic taxa were assessed under the TIPAs Uganda project; other significant contributors of Red List assessments for the Ugandan flora include the Lake Victoria Basin project (East Africa Plant Red List Authority 2019) and the Global Tree Assessment (Cannon et al. 2023).

## ﻿Results

### ﻿Summary

The checklist of the endemic and near-endemic vascular flora of Uganda is presented in Suppl. material 2. Uganda has 52 strict-endemic species and a further 15 strict-endemic infraspecific taxa. Using our definition, it has another 82 species and 35 infraspecific taxa that are near-endemic, hence having a total of 184 endemic and near-endemic taxa (Table 1) in 49 families and 103 genera. In addition, there are 19 undescribed taxa that are potentially endemic or near-endemic to Uganda but for which there is insufficient material available to confirm their taxonomic status. Three hybrid species are also considered to be endemic or near-endemic (Table 1). From here-on, for brevity, the combined strict endemic and near-endemic flora will be referred to as the endemic flora of Uganda.

**Table 1.** Summary of the endemic flora of Uganda.

Taxon rank	Uganda strict-endemics	Uganda near-endemics	Uganda strict-endemics and near-endemics
Species	52	82	134
Subspecies	5	18	23
Variety	10	17	27
**Total described taxa**	**67**	**117**	**184**
Undescribed taxa	18	1	19
Hybrids	2	1	3
**Total taxa**	**87**	**119**	**206**

Table 2 provides a breakdown of the most taxon-rich plant families within the endemic and total floras of Uganda. For strict-endemics, Asparagaceae is the most species-rich, with ten species all of which are Dracaena (Sansevieria), whilst joint second are Asphodelaceae and Asteraceae with six species each, the former of which is accounted for by six endemic species of *Aloe* L. With inclusion of near-endemic species, the most species-rich family is Asteraceae with 32 species, double the number of the second most species-rich family, Asphodelaceae, which is again dominated by *Aloe* species. When compared to the total vascular flora of Uganda (J. Kalema, unpubl. data), seven of the ten most species-rich families are shared with the endemic flora, the exceptions being Asphodelaceae, Asparagaceae and Rosaceae which do not feature in the most species-rich families in Uganda as a whole. Of particular note, Poaceae is the second most species-rich family in Uganda but has only one strict-endemic and two near-endemic species, whilst the Fabaceae is the most species-rich family in Uganda but only joint-seventh in terms of richness in endemism.

**Table 2.** Important plant families for published endemic and near-endemic taxa in Uganda (hybrids and unpublished taxa excluded).

Uganda strict-endemics		Uganda strict-endemics and near-endemics		Total vascular flora of Uganda	
1. Asparagaceae	10	1. Asteraceae	32	1. Fabaceae	519
= 2. Asphodelaceae	6	2. Asphodelaceae	16	2. Poaceae	448
= 2. Asteraceae	6	3. Asparagaceae	13	3. Asteraceae	337
= 2. Cyperaceae	6	4. Orchidaceae	11	4. Orchidaceae	307
5. Orchidaceae	5	= 5. Cyperaceae	10	5. Rubiaceae	250
6. Euphorbiaceae	4	= 5. Rubiaceae	10	6. Lamiaceae	172
= 7. Brassicaceae	3	7. Fabaceae	9	7. Euphorbiaceae	148
= 7. Fabaceae	3	8. Rosaceae	8	8. Acanthaceae	146
= 7. Rubiaceae	3	9. Acanthaceae	7	9. Cyperaceae	141
= 7. Zamiaceae	3	10. Euphorbiaceae	6	10. Apocynaceae	101

The 10 plant families with the highest number of endemic taxa, with comparison to the ten most species-rich plant families for the total Ugandan flora. Numbers refer to number of taxa; where two or more plant families share the same number of taxa, the “=” symbol is used to denote that these families have an equal standing in the table.

**Figure 1.**
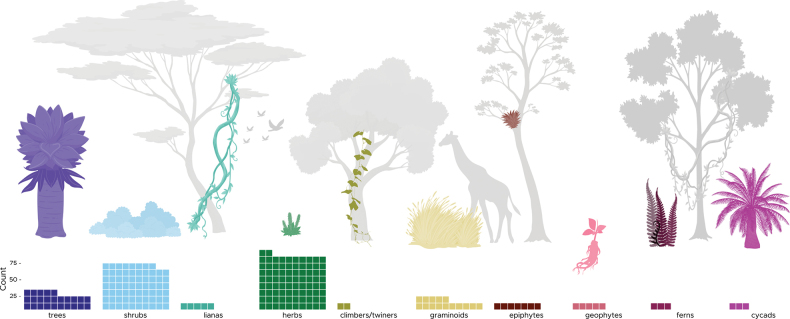
Growth form diversity of the endemic and near-endemic plants of Uganda Note that species can fall under more than one growth form category or sub-category; “count” refers to number of taxa.

The endemic flora of Uganda is represented by a wide range of plant growth forms (Fig. 1). Tree taxa make up only 13.6% of the total endemic flora, with 25 taxa, whilst herbaceous taxa (111) represent 60.3% of the same, the majority (86.5%) being non-graminoid perennials. Only three endemic pteridophytes and three endemic cycads are recorded.

### ﻿History of discovery of the endemic flora

**Figure 2.**
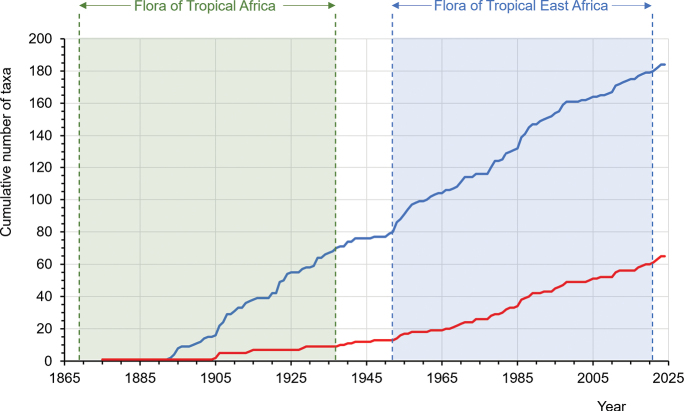
The history of first description of the strict-endemic (red line) and endemic and near-endemic (blue line) flora of Uganda.

The cumulative total (184) of the currently accepted endemic flora of Uganda described over time is shown in Fig. 2. The earliest species to be described was *Alchemilla stuhlmannii* Engl. in 1893, a Rwenzori Mountains endemic on the border of Uganda and the Democratic Republic of the Congo (Rotton et al. 2023a). None of the endemics had been described prior to the onset of the Flora of Tropical Africa series. The description of the endemics has been relatively steady since the 1890s, with only minor accelerations and decelerations occurring up to the present day, including during the writing of FTEA. There has been no noticeable tail-off in endemic species description.

Suppl. material 3 provides a breakdown of the most prolific (a) collectors of type specimens of the endemic flora and (b) authors of these taxa. William Eggeling (1909–1994), and Richard Dummer (or Dümmer; 1887–1922) are the only collectors to have contributed ten or more type collections. In terms of authorship, the number one spot is taken by the contemporary Uganda-based botanist Tom Forrest (1943–), having authored or co-authored 14 of Uganda’s endemic taxa, whilst Bernard Verdcourt (1925–2011), by far the most prolific author of the FTEA series (Beentje 2016), is second with 13 taxa.

### ﻿Geographic distribution

**Figure 3.**
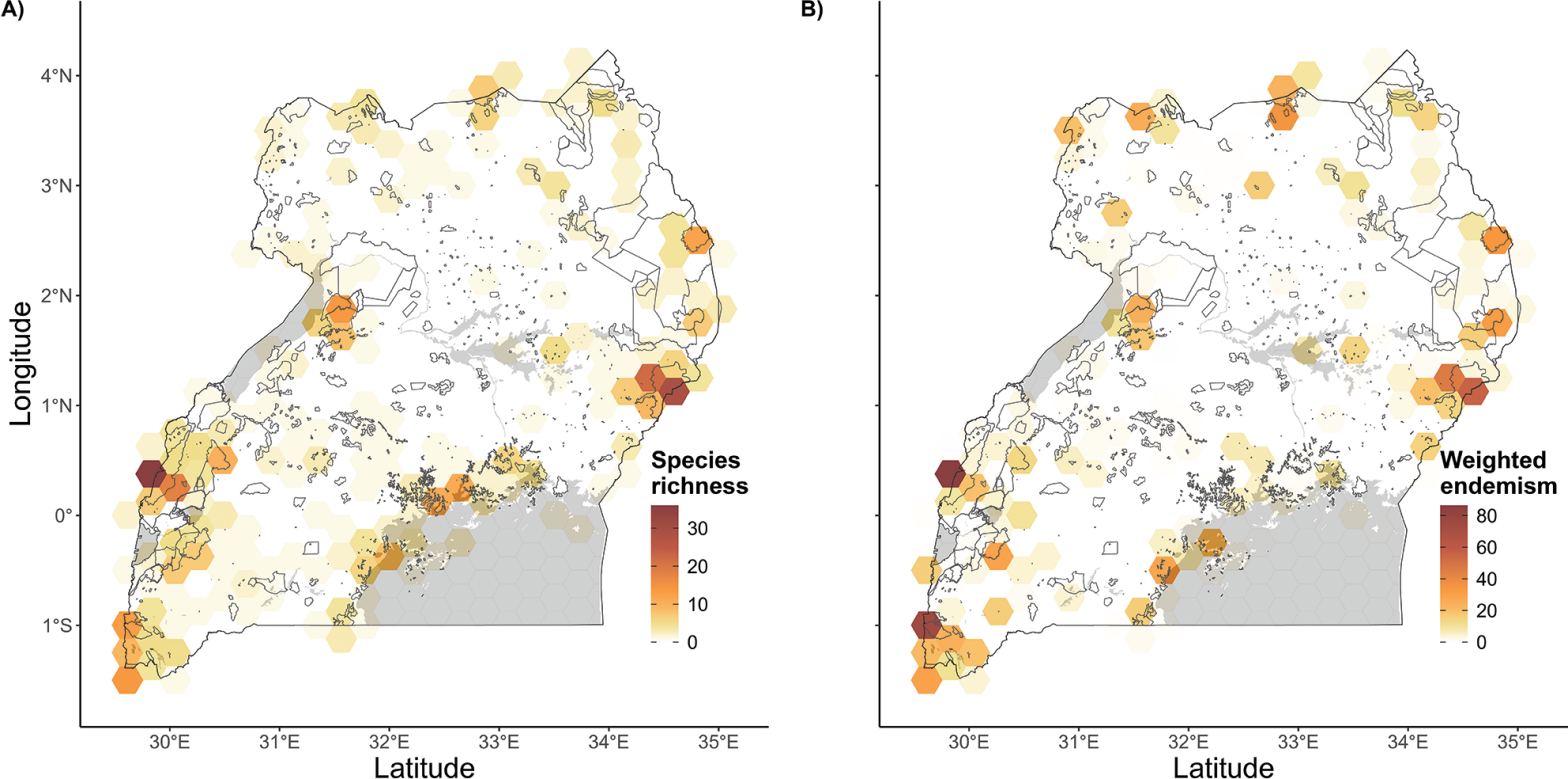
**A.** Richness in endemic and near-endemic taxa, including species, subspecies and varieties; **B.** Total weighted endemism values for each taxon present within a cell.

Endemic taxon richness and weighted endemism are mapped in Fig. 3. These both show concentrations in and adjacent to the Albertine Rift of western Uganda, as well the mountains of eastern Uganda and, to a lesser extent, the fringes of Lake Victoria. The species richness map (Fig. 3A) shows that the richest cells fall within the Rwenzori Mountains and Mount Elgon, accounting for the six highest cells between them. Similar to species richness, Mount Elgon and the Rwenzori Mountains represent the highest values of weighted endemism (Fig. 3B). Cells such as those located in Bwindi-Impenetrable, Mount Moroto, Mount Kadam and Agoro-Agu rank notably higher for weighted endemism than for species richness. The top 10 richest cells and the top 10 cells with highest values for weighted endemism are listed in Table 3.

**Table 3.** Ten taxon-richest cells, including species, sub-species and varieties, alongside the number of endemic taxa recorded in each cell; compared to the sites with the highest weighted endemism values.

Most taxon-rich cells			Highest weighted endemism values		
1.	Rwenzori Mountains	33	1.	Mount Elgon	99.91
2.	Mount Elgon	30	2.	Rwenzori Mountains	90.19
3.	Rwenzori Mountains	29	3.	Bwindi-Impenetrable	79.31
4.	Mount Elgon	23	4.	Rwenzori Mountains	59.00
5.	Rwenzori Mountains	19	5.	Mount Moroto	46.82
6.	Mount Elgon	18	6.	Budongo	42.99
7.	Mgahinga-Gorilla to Bufumbira	15	7.	Mount Kadam	42.67
8.	Budongo	14	8.	Bwindi-Impenetrable	38.63
9.	Mount Moroto	12	9.	Mgahinga-Gorilla to Bufumbira	36.73
10.	Bwindi-Impenetrable	11	10.	Agoro-Agu	35.05

**Figure 4.**
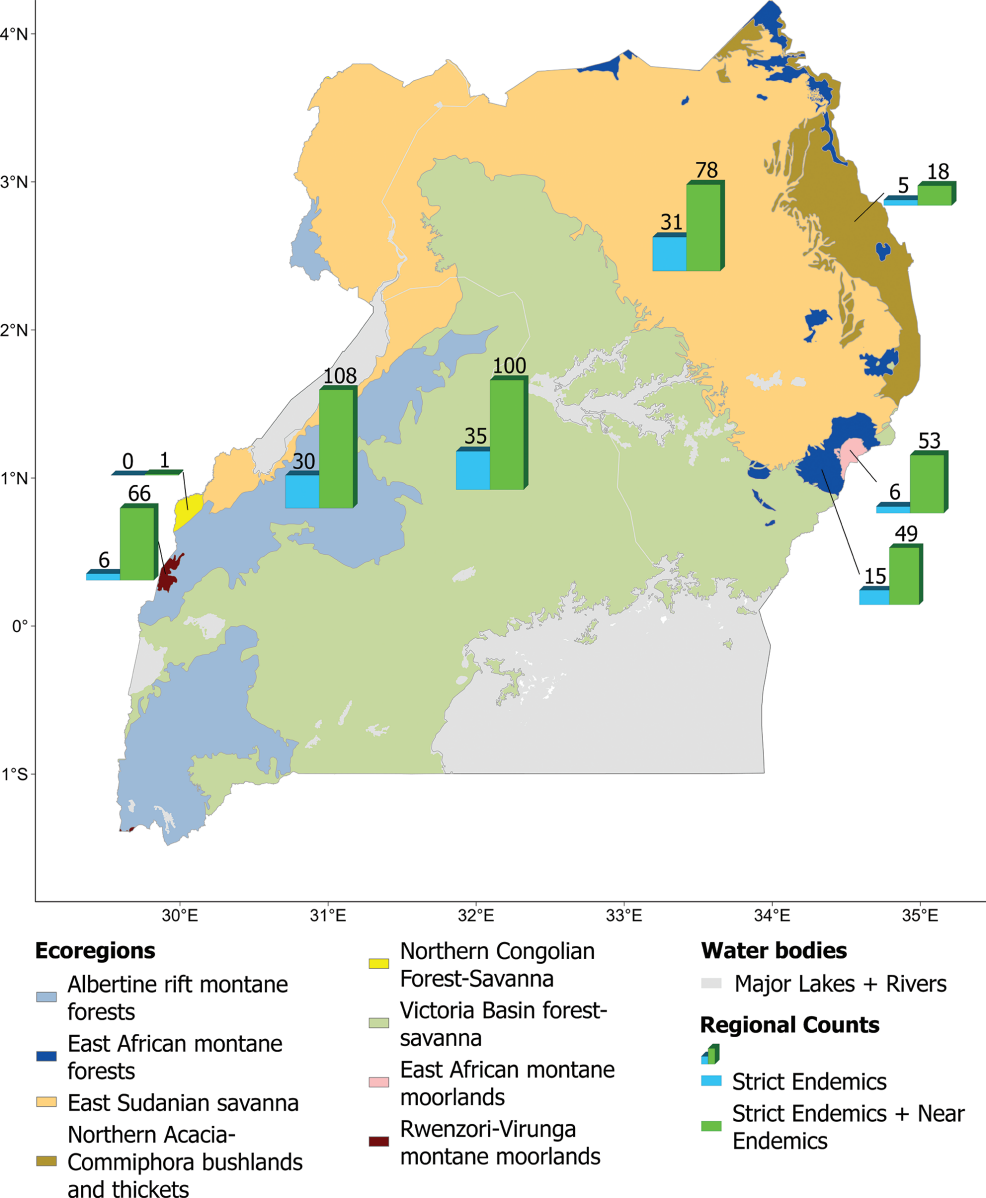
Ecoregions of Uganda and their numbers of strict endemic and near-endemic vascular plant taxa.

At the ecoregion level, plant endemism occurs in all eight ecoregions recorded in Uganda (Fig. 4), but with highly varying importance (Table 4). The four montane ecoregions (Albertine rift and East African montane forests, Rwenzori-Virunga and East African montane moorlands) together account for 122 (66.3%) of the Ugandan endemic flora despite comprising only 13.16% of the total land area of Uganda, and each has between 17 and 29 endemics unique to that ecoregion. The Victoria Basin forest-savanna ecoregion also has a significant number of endemics (38, or 20.7%). On the other hand, the number of endemics is surprisingly low for the Northern *Acacia*-*Commiphora* bushlands and thickets in the northeast of Uganda, with only eight (4.3%) species.

**Table 4.** Summary of the geographic distribution of published endemic taxa in the ecoregions of Uganda. Figures in brackets represent number of taxa unique to that ecoregion in Uganda.

Ecoregion number (after Olson et al. 2001)	Ecoregion	Area in Uganda (km^2^) (percentage of total land area)	Uganda strict-endemics	Uganda strict-endemics and near-endemics
1	Albertine rift montane forests	103,900 (11.18%)	17 (13)	49 (29)
8	East African montane forests	65,500 (1.72%)	9 (6)	27 (17)
43	East Sudanian savanna	917,630 (29.59%)	16 (15)	28 (20)
51	Northern *Acacia-Commiphora* bushlands and thickets	326,000 (3.81%)	3 (2)	8 (5)
52	Northern Congolian Forest-Savanna	703,010 (0.16%)	0 (0)	1 (0)
61	Victoria Basin forest-savanna	165,800 (39.17%)	19 (16)	38 (27)
78	East African montane moorlands	3,089 (0.14%)	4 (2)	28 (19)
86	Rwenzori-Virunga montane moorlands	516 (0.12%)	3 (3)	36 (24)

Similarly, at the level of phytochorion (Suppl. material 4), the Afromontane Archipelago-like Centre of Endemism is by far the most important in Uganda for endemics with 106 taxa (57.6%). Consistent with ecoregions, the Somali-Masai regional centre of endemism (RCE) had a low level of plant endemism, with only eight taxa (4.3%), while the Guinea-Congolia/Sudania regional transition zone (RTZ) registered no single endemic taxon.

### ﻿Extinction risk and conservation status

**Figure 5.**
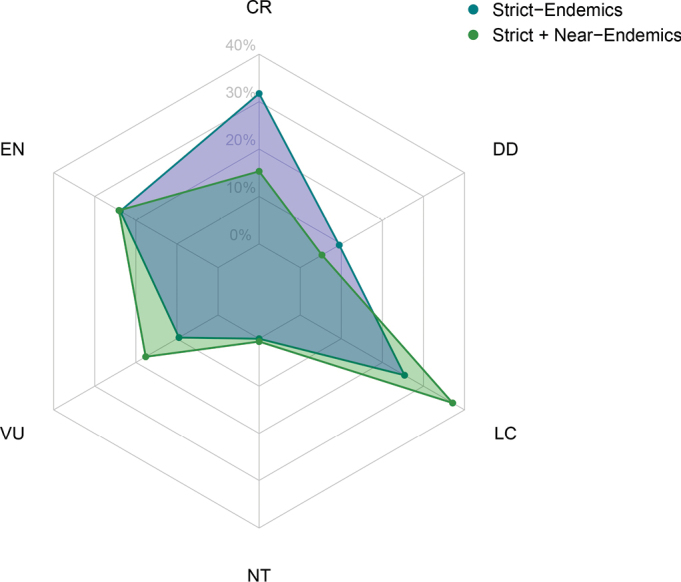
Radar plot of extension risk in the endemic flora of Uganda. For each of the IUCN Red List categories (LC = Least Concern; NT = Near Threatened; VU = Vulnerable; EN = Endangered; CR = Critically Endangered; DD = Data Deficient), the values are given as a percentage of the total endemic flora that has been assessed.

A total of 170 taxa (92.4%) of the endemic flora of Uganda have extinction risk assessments for the IUCN Red List (Suppl. material 5). Of these, 58% are threatened with extinction, this figure rising to 66% when only strict-endemics are considered. On the other hand, 37% of the endemic taxa are considered to be of Least Concern, although this figure falls to 25% for strict-endemics (Fig. 5).

## ﻿Discussion

The total number (134 spp.) of Uganda’s described endemic and near-endemic flora represents 2.78% of the national plant richness of 4,816 species (Beentje 2016), whilst strict endemics are only 1.07% (52 spp.) of the national flora richness. In comparison, Ethiopia has at least 6,027 vascular plant species, of which 544 (9.03%) are strict country endemics (Demissew et al. 2021), while Kenya has 6,596 species of which 467 (7.08%) are strict endemics (Beentje 2016; Zhou et al. 2017) and Tanzania has 12,383 species of which 1,333 (10.77%) are strict endemics (Madoffe et al. 2006). Uganda, therefore, has a comparatively much lower level of plant (strict and near-) endemism than other countries in the East Africa region. This is probably because it is at the edge-of-range for many of the phytochoria and habitat types rather than being within core centres of endemism, with the exception of the montane regions (see below).

### ﻿The most taxon-rich plant families and genera in the endemic flora

Families and genera of plants that are most taxon-rich within the endemic flora vary from one region to another. For instance, in Mozambique, important families for endemic and near-endemic taxa include Fabaceae, Rubiaceae and Euphorbiaceae s.s. (Darbyshire et al. 2019), whilst in East Africa Beentje et al. (2006) report the families with the highest number of narrow endemics in East Africa as Rubiaceae and Fabaceae, and those with the largest percentage of narrow endemics as Asphodelaceae (Aloaceae) and Balsaminaceae. For Ugandan strict-endemics, Asparagaceae is the most endemic-rich family. All ten endemic Asparagaceae belong to the genus Dracaena (Sansevieria), a group that has been subject to recent concerted collection effort and taxonomic study in Uganda (e.g., Forrest 2023; Forrest and Cole 2024), which may account for its prominence in Uganda’s endemic flora. Similarly, the joint second highest family for strict endemics is Asphodelaceae with six species, all of which is accounted for by six endemic species of *Aloe*, another genus that has been subject to detailed recent taxonomic study in Uganda (e.g., Cole and Forrest 2011, 2017). With inclusion of near-endemic species, by far the most species-rich family is Asteraceae with 32 species. This largely reflects the importance of this family in the high montane flora of the Albertine Rift and the mountains of eastern Uganda. These montane regions support high numbers of endemic species; the reasons behind this are discussed in the section on geographic distribution below.

### ﻿Growth forms and habitats of the endemic flora

Uganda’s endemic flora falls within a wide range of growth forms, owing to a variety of habitats in a wide range of altitude ranging from 600 to > 5,000 m a.s.l. (Kalema and Beentje 2012). The majority (>60%), however, have herbaceous growth forms, while c. 42% are woody (the small overlap being because some species have perennial herbaceous and shrubby forms). This is partly because of the endemic-rich families Asphodelaceae, Asparagaceae and Asteraceae which are dominated by herbaceous species. At the other end of the scale, only four species of cycads are known from Uganda, three of which are strictly endemic (Kalema and Beentje 2012). This non-uniform distribution of growth forms is not unusual. In the Tropical East Africa region more generally, for example, new and newly recorded taxa were also found to be diverse, with herbs accounting for the highest percentage (48.4%), followed by shrubs (26.4%), trees (12.2%), lianas (8. 8%), while ferns (2.5%), and cycads (1.8%) also registered the lowest proportions (Du et al. 2023).

Rocky sites are amongst the habitats known to harbour high numbers of endemic plant species in many parts of the world (Darbyshire et al. 2019; García et al. 2019), and this is also true in Uganda. The three endemic cycads occur in rocky habitats at Mpanga Gorge, Buwerere and Era-Lama. The endemic-rich genera *Aloe* and Dracaena (Sansevieria) also typically occur in rocky environments such as at Mubende-Kyegegwa, Tororo and Kachumbala rocks, and the dry montane regions of Karamoja. Such environments provide important unique micro-habitats in stressful ecosystems that are inaccessible to humans and livestock, thus safe sites for the relic and endemic floras (Rafiee et al. 2022; Shakdofa et al. 2024; van der Plas and Olff 2024). Their micro-climates can remain stable for thousands of years, supporting high species diversity and endemism (Fitzsimons and Michael 2017).

### ﻿History of endemic flora discovery in Uganda

Every year, c. 2,500 plant species are named as new to science globally (Antonelli et al. 2023). The value of discovery of new plant species in strengthening our understanding of plant diversity, which in turn supports the identification of areas most in need of protection, has been underscored (e.g., Cheek et al. 2020). However, it takes a long time from encountering to naming a plant as a species new to science, for instance, an average of 16 years for a new tree species in the Neotropics (Antonelli et al. 2023). It is now estimated that three in four undescribed plant species are already threatened with extinction (Brown et al. 2023b). With such a high extinction risk to plants, there is an urgent need to speed up how new species are described and named, lest they are lost before they are described. From 1893, the discovery and description of new endemic taxa in Uganda has continued to rise, with no observable levelling off; this suggests that Uganda’s endemic flora will continue to grow with further targeted surveys of under-explored sites and regions, and that future new discoveries of endemics are likely to already be threatened.

William Eggeling (1909–1994), and Richard Dummer (or Dümmer; 1887–1922) were the most prolific collectors of type specimens of the endemic flora. The former served as a District Forest Officer and later Conservator of Forests in colonial-period Uganda, whilst the latter was employed at Kivuvu Rubber Co. in Uganda, and both were prolific plant collectors (Polhill and Polhill 2015). These placements provided excellent opportunities for both individuals to collect numerous plant specimens, often in previously unbotanised areas, hence a high chance of encountering undescribed endemic taxa.

A slight acceleration in description of endemic taxa is observable after 1952. This is the time the first volume of FTEA was published. Writing of floras involves not only extensive survey of literature and taxonomic examination of herbarium specimens but also regular field surveys to infill geographic gaps in our botanical knowledge (Frodin 2001; Delprete 2014; Sullivan and Nazaire 2021; van Welzen et al. 2024). This created an opportunity to collect and detect endemic taxa. At the same time, writing of other regional floras such as Flora Zambesiaca (1960–present; see Beentje 2016) overlapped for some time with the FTEA project, and these together contributed to a better understanding of subcontinental plant diversity which would also have supported the detection of local endemic taxa.

From the time of completion of the FTEA project in 2012, many new plant species have continued to be discovered in this floristically important region. A total of 54 new taxa, including both species newly described to science and new records in Uganda of previously described species, have been reported to have been discovered in the country since 2012 (Du et al. 2023). Similarly, elsewhere in East Africa, several new species have also been discovered and described from the coastal forests of Kenya (Ngumbau et al. 2020), whilst since completion of the Flora of Ethiopia and Eritrea (FEE) in 2009, nearly 70 indigenous taxa have been described from this area (numbers updated from Demissew et al. 2021). In Uganda, the examples of *Aloe* and Dracaena (Sansevieria) demonstrate that concerted field exploration focussed on specific taxa can also accelerate species discovery. As a further example, recent targeted collections and molecular systematic studies on the genus *Chlorophytum* Ker Gawl. have revealed some potential new species to science in Uganda (P. Matovu & C. Bjorå, pers. comm. 2025).

### ﻿Geographic distribution of the endemic flora

Our understanding of the geographic distribution of Uganda’s endemic flora will become clearer as compilation of plant distribution data becomes more complete and gaps are infilled. This will also help improve conservation strategies, since knowledge gaps of a county’s endemic flora may contribute to its insufficient protection (Carbutt 2023). However, our findings provide a sufficiently comprehensive country-wide picture, with patterns of distribution that are unlikely to change significantly.

High mountains have been found in this study to be centres of endemism in Uganda. Plant endemism is concentrated in, and adjacent to, the upland areas flanking the Albertine Rift in western Uganda, as well the more isolated mountains of eastern Uganda. To this effect, the richest sites for endemic species are the Rwenzori Mountains and Mount Elgon. These two massifs also represent the highest weighted endemism in Uganda. Other important highlands are Bwindi-Impenetrable forest, Mgahinga-Gorilla to Bufumbira, Mount Moroto, Mount Kadam and Agoro-Agu, some of which rank notably higher for weighted endemism than for species richness (Table 3). These montane sites are therefore of considerable conservation importance at both national and global scales. Indeed, all of these sites qualify as Important Plant Areas (J. Kalema et al., in prep.; https://tipas.kew.org/). Tropical high mountains are known to host important hotspots of biodiversity within small, mostly remote areas (Kidane et al. 2019). Unfortunately, some of these Ugandan montane sites, such as Agoro-Agu, are facing severe threats including encroachment for agriculture, settlement and unsustainable extraction of resources (Okullo et al. 2021; Birungi et al. 2023) that need to be addressed urgently to secure their unique biodiversity.

Uganda’s mountain areas contribute to climatic stability by providing refugia (Marshall et al. 2022) and habitat heterogeneity that have resulted in their notable endemism. Being sky islands, the Rwenzori Mts and Mt Elgon represent unique ecosystems that have promoted allopatric divergence through a long period of isolation, thus enhancing endemism. Such phenomena have also been observed by Oswald et al. (2020) in the mountains of the Cape provinces of South Africa, and by Dagallier et al. (2023) in the broader tropical African mountains.

The only non-montane site that falls within the 10 most endemic taxon-rich cells and the 10 highest cells for weighted endemism is the Budongo Central Forest Reserve, a well-known mid-elevation moist semi-deciduous forest with Guineo-Congolian forest elements, including being dominated by one species of Leguminosae: in this case *Cynometra alexandri* C.H.Wright (White 1983; also see Langdale-Brown et al. 1964; van Breugel et al. 2015). This is one of the most extensively botanised sites in Uganda, yet it may still reveal further endemic taxa – for example, the strict-endemic species *Afrothismia ugandensis* is known only from two collections to date despite occurring in the most densely surveyed section of the reserve (Cheek et al. 2024b).

Whilst the four montane ecoregions (Albertine rift, East African montane forests, Rwenzori-Virunga and East African montane moorlands) are the richest in endemics, all eight ecoregions of Uganda support endemic taxa. The Victoria Basin forest-savanna ecoregion, in particular, also registered a significant number of endemics. The basin encompasses most of south-central Uganda and supports a mixture of forest and savanna habitats. Most of the ecoregion receives between 1,000 to 1,400 mm rainfall, exceeding 2,000 mm on the Ssese Islands in Lake Victoria. The Ssese Islands are also important for the conservation of Uganda’s flora, as they contain at least six endemic or highly range-restricted species of conservation concern (Kalema et al. 2025). However, no single site (grid cell) of this ecoregion falls within the richest sites or those with highest weighted endemism (Table 3).

The Northern *Acacia*-*Commiphora* bushlands and thickets in the northeast of Uganda registered a surprisingly low number of endemics with only eight (4.3%) species. This ecoregion is part of the Somali-Masai regional centre of endemism, a phytochorion with high overall levels of plant endemism, particularly within the Horn of Africa Biodiversity Hotspot (Thulin 2004). Karamoja region in Uganda is within this RCE. It is characterised by an arid to semi-arid climate with rainfall that is unreliable and sparsely distributed, causing extreme and recurrent droughts, and at other times flooding (IGAD 2020). The insecurity which characterised this region for decades, fuelled by use of firearms and cattle rustling, made the region unsafe for botanical surveying, thus remaining poorly inventoried to adequately document its flora, including endemic plants. This inadequate survey, coupled with the area being on the edge of this RCE, might contribute to its apparently low level of endemism.

### ﻿Extinction Risk in Uganda’s endemic flora

The world over, more than 70% of all vascular plants lack extinction risk assessments and are not on the IUCN Red List (Bachman et al. 2024). In Uganda, this figure stands at ca. 66%. However, more than 90% of Uganda’s endemic flora has now been assessed for its extinction risk, more than half (58%) of which is threatened with extinction (CR, EN, or VU). For comparison, the corresponding threat figure for the Greater Midlands Centre of Floristic Endemism of South Africa is 60% (Carbutt 2023), whilst in Mozambique 55% of the endemic and near-endemic flora was assessed as threatened (Darbyshire et al. 2023). The endemic flora of Uganda is a key element for setting national conservation priorities and for driving conservation strategies, since its persistence is entirely dependent on national policy (Orsenigo et al. 2018). These taxa therefore need special attention for their conservation, particularly given these high levels of threat.

Uganda’s figures for threatened endemics might be even higher if some of the currently Data Deficient (DD) species turn out to be as threatened, or even more threatened, than those endemics with sufficient data for assessment (Borgelt et al. 2022; Cazalis et al. 2023). The nine (5.3%) endemic taxa we assessed as DD because there was inadequate information about their population status, distribution or threats and their habitats to be assessed for their risk of extinction (Bland et al. 2017; IUCN 2025), require urgent attention. There is a need to conduct targeted surveys of their known sites of occurrence to infill these knowledge gaps. Such taxa are potentially of high conservation concern once enough information becomes available (Bland et al. 2017; Borgelt et al. 2022; Tafesse and Yohannes 2022; Cazalis et al. 2023).

More than one third (36.5%) of Uganda’s endemic flora have been assessed at Least Concern. These need to be maintained as such by implementing conservation plans that ensure ecological integrity of their habitats. Coupled with these should be close monitoring of these taxa and their habitats for early detection of any events or processes that may render them threatened, and for timely corrective measures to avert their endangerment.

Further, many of the endemic taxa currently assessed as Least Concern occur in mountainous areas at high altitude. By occurring in these sky islands, these taxa remain as isolated populations. The projected increase in global temperatures due to climate change is likely to result in more isolation of high-elevation species as lowland habitat expands and the alpine zones shift upslope, with the lower altitudes surrounding them acting as a barrier to dispersal (Oswald et al. 2020). Thus, climate change is likely to increase the extinction risk for montane endemic plant taxa. As climate change has not yet been incorporated into the Red List assessments of Uganda’s endemic flora, it is therefore likely that the level of threat it faces will be even higher than the 57% recorded to date. Indeed, preliminary models predict that many of the Afroalpine endemics of the Rwenzori and Mt Elgon are facing elevated future extinction risk related to projected climate change (S. L. Richards et al., in prep.).

In addition, there are some species in Uganda that do not meet the near-endemic criteria but are still priorities for biodiversity conservation in Uganda, including globally threatened taxa and highly range restricted but disjunct species. For example, *Acanthopale macrocarpa* Vollesen is known only from three localities, Kibale National Park in Uganda, Kakamega Forest in Kenya and a recently discovered population at Cyamudongo in Rwanda (E. Fischer, unpubl. data). Based on the first two subpopulations only, it was assessed as Least Concern due to effective conservation management at its known sites (Luke et al. 2015). Similarly, *Poa chokensis* S.M.Phillips is known only from two localities, Mt Elgon in Uganda and the Choke Mountains of Ethiopia, and is assessed as Endangered (Lulekal et al. 2022), whilst *Leptonychia semlikensis* Engl. is known only from the western border of Uganda and eastern border of the Democratic Republic of the Congo, and is assessed as Vulnerable (Rotton et al. 2023b). Whilst falling short of qualifying as near-endemic, they too have very limited geographical ranges and hence merit conservation attention and strategies to protect them. Their continued survival is heavily dependent on conservation actions by, and a responsibility of, the countries they occur in. Sites of occurrence for these species deserve inclusion in the country’s conservation planning processes. Such “cold spots,” harbour significant plant diversity and should not be overlooked (Carbutt 2023).

The protected area network in Uganda is key to protection of the country’s flora, including the endemics. Dagallier et al. (2020) found a very high (> 85%) overlap between centres of phylogenetic endemism and protected areas. A large proportion of Uganda’s endemic flora are covered within the existing protected area network, but others are not. Limited ecological connectivity, together with environmental degradation and unsustainable utilization, are key factors endangering Uganda’s endemic flora, increasing their risk of extinction. Habitats continue to degrade and are lost owing to the rapid rise in human population size at an average annual growth rate of 2.9% (Uganda Bureau of Statistics 2024), and the associated heightened pressure on natural resources. The Red List of Ecosystems (RLE) was developed by IUCN to identify habitats most at risk of collapse to prioritize the conservation efforts needed to avert such an event. In Uganda, the value of applying RLE criteria at a national scale and how this may influence the IPA network has been assessed by Richards et al. (2024). They identified that eleven of the twenty habitat types identified in Uganda are facing varying levels of risk of collapse nationally, three of which (Lowland bamboo, Evergreen and semi-evergreen bushland and thicket, and Lake Victoria drier peripheral semi-evergreen Guineo-Congolian rain forest) are Critically Endangered. With such high levels of risk to habitats nationally, it is not surprising that many of the endemic species are at high risk of extinction.

In addition to species-based approaches, conservation action through spatial planning is therefore of prime importance. Spatial conservation prioritisation guides conservation effort towards key sites, whilst also ensuring ecological connectivity and gene flow. This maximises survival and continuity of species and enables optimal allocation of scarce resources (Nagkoulis et al. 2025). This is consistent with global conservation targets, such as the “30 by 30” target, in order to ensure the protection of the world’s biodiversity (Fastré et al. 2021). Spatial data and integrated spatial planning help countries to monitor the state of their ecosystems and determine how and where to intervene (UNDP 2023). The threats to Uganda’s plant diversity, and specifically to the endemic flora and their associated habitats, point to an urgent need to plan and implement effective site-based conservation programmes. Safeguarding of this unique endemic flora is pivotal to sustainable management and development.

## ﻿Conclusions

This study provides the first comprehensive account of the endemic flora of Uganda, its distribution and its threat status. Whilst proportionately smaller than the endemic floras of some of its East African neighbours, the 184 endemic and near-endemic plant taxa recorded represent an important subset of Uganda’s biodiversity over which it has a responsibility of stewardship. We find significant concentrations of the endemic flora geographically, particularly in the montane regions bordering the Albertine Rift and in the eastern portion of the country, and these distribution patterns in the endemic flora help to guide the identification of IPAs to optimise the use of limited resources in conservation planning and action. Furthermore, we find that the endemic flora of Uganda is highly threatened, with 58% of the endemics currently assessed as at risk of extinction. As some of the sites harbouring endemic taxa are unprotected at present, there is a great urgency to the effective implementation of the Global Biodiversity Framework in Uganda if its exceptional national biodiversity is to be preserved.

## ﻿Additional information

### Use of AI

No use of AI was reported.

### Author ORCIDs

James Kalema https://orcid.org/0000-0003-4044-3917

Sophie L. Richards https://orcid.org/0000-0002-2466-2522

Samuel Ojelel https://orcid.org/0000-0003-0328-3256

Haley Gladitsch https://orcid.org/0009-0009-5337-1941

Hannah Wheatcroft https://orcid.org/0009-0002-6991-8162

Henry Miller https://orcid.org/0009-0005-5842-7685

W. R. Quentin Luke https://orcid.org/0000-0002-7256-031X

Iain Darbyshire https://orcid.org/0000-0002-5514-9561

### Data availability

All of the data that support the findings of this study are available in the main text or Supplementary Information.

## Supplementary materials

Supplementary material 1 Methodology for weighted endemism analysis Data type docx https://binary.pensoft.net/file/1506141This dataset is made available under the Open Database License (http://opendatacommons.org/licenses/odbl/1.0/). The Open Database License (ODbL) is a license agreement intended to allow users to freely share, modify, and use this Dataset while maintaining this same freedom for others, provided that the original source and author(s) are credited.James Kalema, Sophie L. Richards, Samuel Ojelel, Haley Gladitsch, Hannah Wheatcroft, Henry Miller, Kennedy Mukasa, Ben Kirunda, Thomas G. Forrest, Thomas C. Cole, Isabel Baldwin, Elizabeth Downes, W. R. Quentin Luke, Iain Darbyshire

Supplementary material 2 Annotated checklist of the endemic and near-endemic vascular flora of Uganda Data type xlsx https://binary.pensoft.net/file/1506142This dataset is made available under the Open Database License (http://opendatacommons.org/licenses/odbl/1.0/). The Open Database License (ODbL) is a license agreement intended to allow users to freely share, modify, and use this Dataset while maintaining this same freedom for others, provided that the original source and author(s) are credited.James Kalema, Sophie L. Richards, Samuel Ojelel, Haley Gladitsch, Hannah Wheatcroft, Henry Miller, Kennedy Mukasa, Ben Kirunda, Thomas G. Forrest, Thomas C. Cole, Isabel Baldwin, Elizabeth Downes, W. R. Quentin Luke, Iain Darbyshire

Supplementary material 3 Most prolific collectors of type specimens of endemic and near-endemic taxa in Uganda Data type docx https://binary.pensoft.net/file/1506143This dataset is made available under the Open Database License (http://opendatacommons.org/licenses/odbl/1.0/). The Open Database License (ODbL) is a license agreement intended to allow users to freely share, modify, and use this Dataset while maintaining this same freedom for others, provided that the original source and author(s) are credited.James Kalema, Sophie L. Richards, Samuel Ojelel, Haley Gladitsch, Hannah Wheatcroft, Henry Miller, Kennedy Mukasa, Ben Kirunda, Thomas G. Forrest, Thomas C. Cole, Isabel Baldwin, Elizabeth Downes, W. R. Quentin Luke, Iain Darbyshire

Supplementary material 4 Summary of the geographic distribution of published endemic taxa in the phytochoria of Uganda Data type docx https://binary.pensoft.net/file/1506144This dataset is made available under the Open Database License (http://opendatacommons.org/licenses/odbl/1.0/). The Open Database License (ODbL) is a license agreement intended to allow users to freely share, modify, and use this Dataset while maintaining this same freedom for others, provided that the original source and author(s) are credited.James Kalema, Sophie L. Richards, Samuel Ojelel, Haley Gladitsch, Hannah Wheatcroft, Henry Miller, Kennedy Mukasa, Ben Kirunda, Thomas G. Forrest, Thomas C. Cole, Isabel Baldwin, Elizabeth Downes, W. R. Quentin Luke, Iain Darbyshire

Supplementary material 5 Summary of the extinction risk status of published endemic taxa in Uganda Data type docx https://binary.pensoft.net/file/1506145This dataset is made available under the Open Database License (http://opendatacommons.org/licenses/odbl/1.0/). The Open Database License (ODbL) is a license agreement intended to allow users to freely share, modify, and use this Dataset while maintaining this same freedom for others, provided that the original source and author(s) are credited.James Kalema, Sophie L. Richards, Samuel Ojelel, Haley Gladitsch, Hannah Wheatcroft, Henry Miller, Kennedy Mukasa, Ben Kirunda, Thomas G. Forrest, Thomas C. Cole, Isabel Baldwin, Elizabeth Downes, W. R. Quentin Luke, Iain Darbyshire
