# Supplementary material for: ﻿Uganda’s endemic flora: discovery, diversity, distribution and threat status
Source: PhytoKeys. 2026 Jan 6;269:1–30. doi: 10.3897/phytokeys.269.173801 (PMC12800779; doi:10.3897/phytokeys.269.173801)
Supplement: Supplementary material 1 — Methodology for weighted endemism analysis [file phytokeys-269-001_article-173801__-s001.docx]

**S1. Methodology for Weighted Endemism Analysis**

Weighted endemism (WE) is calculated for each taxon (including species, subspecies and varieties) as the inverse of the range of the species using the following formula:

$$WE=\sum_{i=1}^{SR} {(scaled EOO*scaled AOO)}^{-1}$$

Both Extent of Occurrence (EOO) and Area of Occupancy (AOO) are calculations used in IUCN Red List Assessments. Previous research has found that these measures do not always correlate. EOO represents the geographical span of a species, calculated as the area of a minimum convex hull encompassing all occurrence points, while AOO, number of cells occupied by occurrence points in a 4 km x 4 km grid, can account for rare species within a larger geographical span, for instance species endemic to montane habitats (Guerin et al. 2015). Therefore, the reciprocal of both EOO and AOO were multiplied to calculate weighted endemism to account for both limited geographical spans and rarity as forms of endemism. EOO and AOO was calculated using the *red* package in R (Cardoso and Branco 2023). Where a convex hull could not be generated, as a species had two or fewer occurrence points, EOO was assumed to equal AOO, as the former cannot be smaller than the latter (Akçakaya 2024; IUCN Standards and Petitions Committee 2024). To standardise EOO and AOO values and balance their influence on weighted endemism, as EOO is usually significantly larger than AOO, both values were centred and scaled using the formula:

$$scaled EOO or AOO=\frac{(species EOO or AOO-mean EOO or AOO of all species)}{standard deviation of EOO or AOO for all species}+1$$

Standardising EOO and AOO values resulted in an overall mean of 0 and a standard deviation of 1 for both the EOO and AOO values respectively. To remove negative values, 1 was added to the standardised EOO and AOO.
