# Supplementary material for: ﻿Uganda’s endemic flora: discovery, diversity, distribution and threat status
Source: PhytoKeys. 2026 Jan 6;269:1–30. doi: 10.3897/phytokeys.269.173801 (PMC12800779; doi:10.3897/phytokeys.269.173801)
Supplement: Supplementary material 3 — Most prolific collectors of type specimens of endemic and near-endemic taxa in Uganda [file phytokeys-269-001_article-173801__-s003.docx]

| **Lead collector** | **Number of type specimens of Ugandan endemic and near-endemic taxa collected** | **Author** | **Number of names of Ugandan endemic and near-endemic taxa (co-)authored** |
| --- | --- | --- | --- |
| 1. W. Eggeling | 13 | 1. T. Forrest | 14 |
| 2. R. Dümmer | 10 | 2. B. Verdcourt | 13 |
| 3. T. Forrest | 9 | 3. C. Jeffrey | 9 |
| 4. J. Purseglove | 7 | = 4. T. Cole | 7 |
| 5. G. Reynolds | 6 | = 4. S. Moore | 7 |
| = 6. T. Cole | 5 | = 6. S. Carter | 6 |
| = 6. O. Hedberg | 5 | = 6. K. Lye | 6 |
| = 8. A. Bagshawe | 4 | = 6. V. Summerhayes | 6 |
| 9.= J. Snowden | 4 | 9. G. Mildbraed | 5 |
| 9= E. Tweedie | 4 |  |  |

**Table S3.** (a) Most prolific collectors of type specimens of endemic and near-endemic taxa in Uganda (only types collected in Uganda are considered, and only lead collectors are listed), and (b) most prolific authors of Ugandan endemic and near-endemic taxa.
