# Supplementary material for: ﻿Uganda’s endemic flora: discovery, diversity, distribution and threat status
Source: PhytoKeys. 2026 Jan 6;269:1–30. doi: 10.3897/phytokeys.269.173801 (PMC12800779; doi:10.3897/phytokeys.269.173801)
Supplement: Supplementary material 4 — Summary of the geographic distribution of published endemic taxa in the phytochoria of Uganda [file phytokeys-269-001_article-173801__-s004.docx]

| **Phytochorion number (after White 1983)** | **Phytochorion** | **Uganda strict-endemics** | **Uganda strict-endemics and near-endemics** |
| --- | --- | --- | --- |
| I | Guineo-Congolian RCE | 11 (7) | 24 (10) |
| III | Sudanian RCE | 16 (15) | 27 (19) |
| IV | Somalia-Masai RCE | 3 (2) | 8 (6) |
| VIII | Afromontane Archipelago-like CE | 21 (18) | 106 (92) |
| XI | Guinea-Congolia/Sudania RTZ | 0 (0) | 0 (0) |
| XII | Lake Victoria RM | 18 (17) | 37 (28) |

**Table S4.** Summary of the geographic distribution of published endemic taxa in the phytochoria of Uganda (White 1983). CE = centre of plant endemism; RCE = regional centre of plant endemism; RM = regional mosaic; RTZ = regional transition zone. Figures in brackets are number of taxa unique to that phytochorion in Uganda.
