# Supplementary material for: ﻿Uganda’s endemic flora: discovery, diversity, distribution and threat status
Source: PhytoKeys. 2026 Jan 6;269:1–30. doi: 10.3897/phytokeys.269.173801 (PMC12800779; doi:10.3897/phytokeys.269.173801)
Supplement: Supplementary material 5 — Summary of the extinction risk status of published endemic taxa in Uganda [file phytokeys-269-001_article-173801__-s005.docx]

|  | **Uganda strict-endemics** | | **Uganda strict-endemics and near-endemics** | |
| --- | --- | --- | --- | --- |
| **IUCN Red List Category** | **Number of taxa** | **% of taxa** | **Number of taxa** | **% of taxa** |
| Total taxa assessed | 64 | 95.5% | 170 | 92.4% |
| CR | 21 | 32.8% | 26 | 15.3% |
| EN | 15 | 23.4% | 42 | 24.7% |
| VU | 6 | 9.4% | 30 | 17.6% |
| NT | 0 | 0% | 1 | 0.6% |
| LC | 16 | 25.0% | 62 | 36.5% |
| DD | 6 | 9.4% | 9 | 5.3% |
| Total threatened | 42 | 65.6% | 98 | 57.6% |

**Table S5.** Summary of the extinction risk status of published endemic taxa in Uganda.

The “% of taxa” figure for “Total taxa assessed” is given as a percentage of all the strict-endemic (left) and endemic plus near-endemic (right) taxa listed in Appendix 1; for each of the Red List categories (LC = Least Concern; NT = Near Threatened; VU = Vulnerable; EN = Endangered; CR = Critically Endangered; DD = Data Deficient), the “% of taxa” is given as a percentage of those taxa that have been assessed.
